# High-resolution computed tomography findings in young infants with cystic fibrosis detected by newborn screening

**DOI:** 10.6061/clinics/2019/e1399

**Published:** 2019-10-14

**Authors:** Renata Wrobel Folescu Cohen, Tânia Wrobel Folescu, Marcia Cristina Bastos Boechat, Vania Matos Fonseca, Elizabeth Andrade Marques, Robson Souza Leão

**Affiliations:** I Faculdade de Ciencias Medicas, Universidade do Estado do Rio de Janeiro, Rio de Janeiro, RJ, BR; II Instituto Nacional de Saude da Mulher, da Crianca e do Adolescente Fernandes Figueira, Fundacao Instituto Oswaldo Cruz, Rio de Janeiro, RJ, BR

**Keywords:** Lung Diseases, Cystic Fibrosis, Neonatal Screening, Tomography, Microbiology

## Abstract

**OBJECTIVE:**

High-resolution computed tomography (HRCT) allows the early detection of pathological changes in the lung structure, and reproducible scoring systems can be used to quantify chest computed tomography (CT) findings in patients with cystic fibrosis (CF). The aim of the study was to describe early HRCT findings according to a validated scoring system in infants with CF diagnosed by newborn screening (NBS).

**METHODS:**

This cross-sectional study included infants with CF diagnosed by NBS who were born between January 2013 and January 2017 and who underwent HRCT scanning within the first year after diagnosis when they were clinically stable. The CT scans were evaluated using the modified Bhalla score.

**RESULTS:**

Thirty-two subjects underwent HRCT scanning. The mean total-modified Bhalla score was 3.6±2.1, and 93.8% of the scans were abnormal. *Pseudomonas aeruginosa* airway colonization was associated with increased modified Bhalla score values. Bronchial wall thickening was the most common feature (90.6%), followed by bronchial collapse/consolidation (59.4%), mosaic attenuation/perfusion (50%), bronchiectasis (37.5%) and mucus plugging (15.6%). Bronchial wall thickening was diffuse in most of the patients.

**CONCLUSION:**

A substantial proportion of infants diagnosed with CF after detection by NBS already showed evidence of lung disease. *P. aeruginosa* colonization was associated with increased Bhalla scores, highlighting the importance of this CF pathogen in early structural lung disease. The presence of bronchial wall thickening at such a young age may reflect the presence of airway inflammatory processes. The detection and quantification of structural abnormalities with the modified Bhalla score may aid in the identification of lung disease before it is clinically apparent.

## INTRODUCTION

Newborn screening (NBS) for cystic fibrosis (CF) has been introduced in many parts of the world, including Brazil, with the aim of obtaining a CF diagnosis as early as possible (1-3). The prognosis for lung health has improved dramatically with the implementation of NBS for CF. Since respiratory disease has been well established as the major cause of morbidity and mortality in children with CF (4), understanding the factors responsible for the initiation and progression of lung disease in early life is of utmost importance (5). Dysfunction in the expression of CF transmembrane regulator (CFTR) leads to a wide and variable array of presenting manifestations and complications. As a general rule, mutations that are considered severe are almost always associated with high sweat chloride values and pancreatic insufficiency, but the pulmonary phenotype is greatly influenced by other factors (6). Patients initially develop transient *Pseudomonas aeruginosa* infection that can be eradicated, but subsequent chronic infection with *P. aeruginosa* or other pathogens, such as those in the *Burkholderia cepacia* complex, is usually associated with impaired lung function (7), poor nutritional status and decreased survival in CF patients (8,9).

Although respiratory manifestations can be diagnosed clinically by a medical history and physical examination along with an assessment of growth parameters, these indicators are relatively insensitive for lung disease evaluations (10). It is well known that pulmonary function tests and chest X-rays are relatively insensitive for indicators of lung disease, such as early and localized lesions, especially in young children (11). Several studies have found that chest computed tomography (CT) images are more sensitive than chest X-rays in detecting lung disease in CF patients (12-13).

The development of high-resolution computed tomography (HRCT) has allowed the early detection of pathological changes in lung parenchyma as well as in the proximal and distal airways (bronchial wall thickening, bronchiectasis, mucoid impaction and lung hyperinflation) (11). Reproducible scoring systems can be used to quantify CT findings (14,15). Furthermore, some studies that used HRCT scoring systems for children and adults with CF and well-established lung disease demonstrated more severe bronchiectasis in the upper lobes (16-18). Although there have been studies investigating the application of various CT scoring systems, a formal recommendation for the routine use of such systems has yet to be made for CF patients (19). In this scenario, the modified Bhalla CT scoring system has been shown to be reproducible and reliable for evaluating HRCT scans, even in patients with mild CF lung disease (14).

The frequency and distribution of HRCT findings in infants with CF lung disease detected by NBS is not yet fully understood. Since the application of CF therapies is increasing in young children, objective measures that allow the easy quantification and represent the distribution of lung damage will be useful not only for this purpose but also for initiating prompt interventions when needed.

The aim of the study was to describe early HRCT findings according to a validated scoring system in young children with CF diagnosed by NBS.

## MATERIALS AND METHODS

This cross-sectional study was a part of a cohort evaluation conducted at the National Institute of Women, Children and Adolescents Health Fernandes Figueira (IFF), which is a technical-scientific unit of the Oswaldo Cruz Foundation (FIOCRUZ) that is focused on education, research, service, technological development and extension in the health of women, children and adolescents. IFF/FIOCRUZ is the main pediatric CF center located in the city of Rio de Janeiro, Brazil. The study was approved by the Research Ethics Committee of the National Institute of Women, Children and Adolescents Health Fernandes Figueira (IFF/FIOCRUZ) (CAAE 58291616.7.0000.5269).

All of the patients with CF-positive NBS tests in Rio de Janeiro state are referred to this CF center to confirm or reject the diagnosis. Once CF is confirmed, subjects are followed until they are 18 years old by a multidisciplinary team. All infants with CF diagnosed by NBS born between January 2013 and January 2017 were included in this study. The medical records of those infants were reviewed.

At our CF center, the current follow-up strategy consists of HRCT examinations beginning the first year after diagnosis when the patients are clinically stable from a respiratory standpoint; stability is defined as the absence of exacerbation criteria (20,21). All HRCT scans were performed with a 16-MDCT scanner (Bright Speed Elite™, General Electric, Milwaukee, WI, USA) with the following parameters: 100 kV, 60-80 mA, gantry rotation time 0.8 seconds per slice and collimation 0.63 mm. This protocol routinely used in the radiology department of the institution is in accordance with the literature (13,22,23). All of the patients were scanned without sedation and during spontaneous breathing. All CT images were evaluated with lung window settings (level, -700 HU; window, 1500 HU) and assessed by an experienced pediatric radiologist who was blinded to the children’s clinical data, clinical history and microbiology data. The HRCT scoring system used in the present study was the modified Bhalla CT score (24). Regarding the distribution, each scan was divided into 6 lobes, with the lingula considered as a separate lobe. The total score for each patient was obtained by summing the scores for each morphological change, which were attributed based on the severity/extent of the abnormality. Scores from 0-3 were assigned to each of the following categories: the severity of bronchiectasis, presence of peribronchial thickening, extent of bronchiectasis, extent of mucous plugging, presence of sacculations, degree of bronchial involvement, number of bullae, presence of intralobular septal thickening and presences of ground-glass infiltrate. Scores from 0-2 were assigned for emphysema, collapse/consolidation, mosaic attenuation/perfusion pattern, air trapping and acinar nodule (24). The total score could range from zero (absence of abnormalities) to 37 (all abnormalities present and severe). The modified Bhalla score was previously validated in CF patients in another study at this institution (14) and was subsequently established not only for clinical studies but also for patient follow-up (15,25).

For the descriptive analyses, mean values and their standard deviations (SDs) were presented for the continuous variables. The categorical variables were described using absolute and percentage frequencies. Student’s t-test and analysis of variance (ANOVA) were used to compare the variables with normal distributions. Nonparametric Mann-Whitney and Kruskal-Wallis tests were used to compare the numerical and categorical variables that had 2 or 3 categories, respectively. The normality assumption was verified with the Shapiro-Wilk test. The analyses were conducted using SPSS software (version 22) (26), and the significance level was 5%.

## RESULTS

Out of 200 patients who were followed in this CF center, 37 patients fulfilled the inclusion criteria (diagnoses obtained from NBS), and 35 were included in the study (2 subjects moved to another state).

The majority of patients were male (n=23; 65.7%), and the mean age at diagnosis was 3.8 months. The z-score for weight-for-length at the moment of CF diagnosis was -0.86 (SD±1.5). Regarding prenatal and newborn data, the mean gestational age was 38.6 weeks (SD±1.1), and only one patient presented a premature delivery (35 weeks). The mean weight at birth was 3056.6 g (SD±456 g). The genotypes for thirty-one patients were available, and F508del was present in 61.2% of the included patients (32.2% homozygous/29% heterozygous), while 38.7% presented other CFTR mutations. Regarding colonization, 51.4% of the patients were free of CF pathogens, while *P. aeruginosa* was present in 6 infants (17.1%). Seven of the patients (20%) were completely asymptomatic at the time of CF diagnosis. Respiratory signs (defined as tachypnea, abnormal auscultation, cyanosis or digital clubbing) were found in 45.7% of the patients. Cyanosis and digital clubbing were absent among all of the patients. Twenty-three of the patients (65.7%) presented gastrointestinal signs/symptoms, including diarrhea, pallor or jaundice. After the initial laboratory evaluation, which included the fecal elastase level, pancreatic insufficiency was detected in 27 of the patients (77.1%) (Table 1).

Among the 35 patients, 32 (91.4%) underwent HRCT, and 3 died before the exam (2 from CF complications and 1 from an accident). HRCT was conducted in patients at an average of 8.9 months old.

The mean total modified Bhalla score was 3.6±2.1. Overall, 93.8% of the patients presented abnormal parameters. Only two patients (6.3%) presented HRCT scans with normal parameters (scores of 0).

There were no statistically significant associations between the mean modified Bhalla score and the age at the time of CT, genotype, pancreatic sufficiency or overall symptoms. When comparing the *P. aeruginosa*-colonized and noncolonized subgroups, the former presented an increased modified Bhalla score, with a statistically significant difference (*p*-value 0.048) (Table 2).

**Figure 1:**
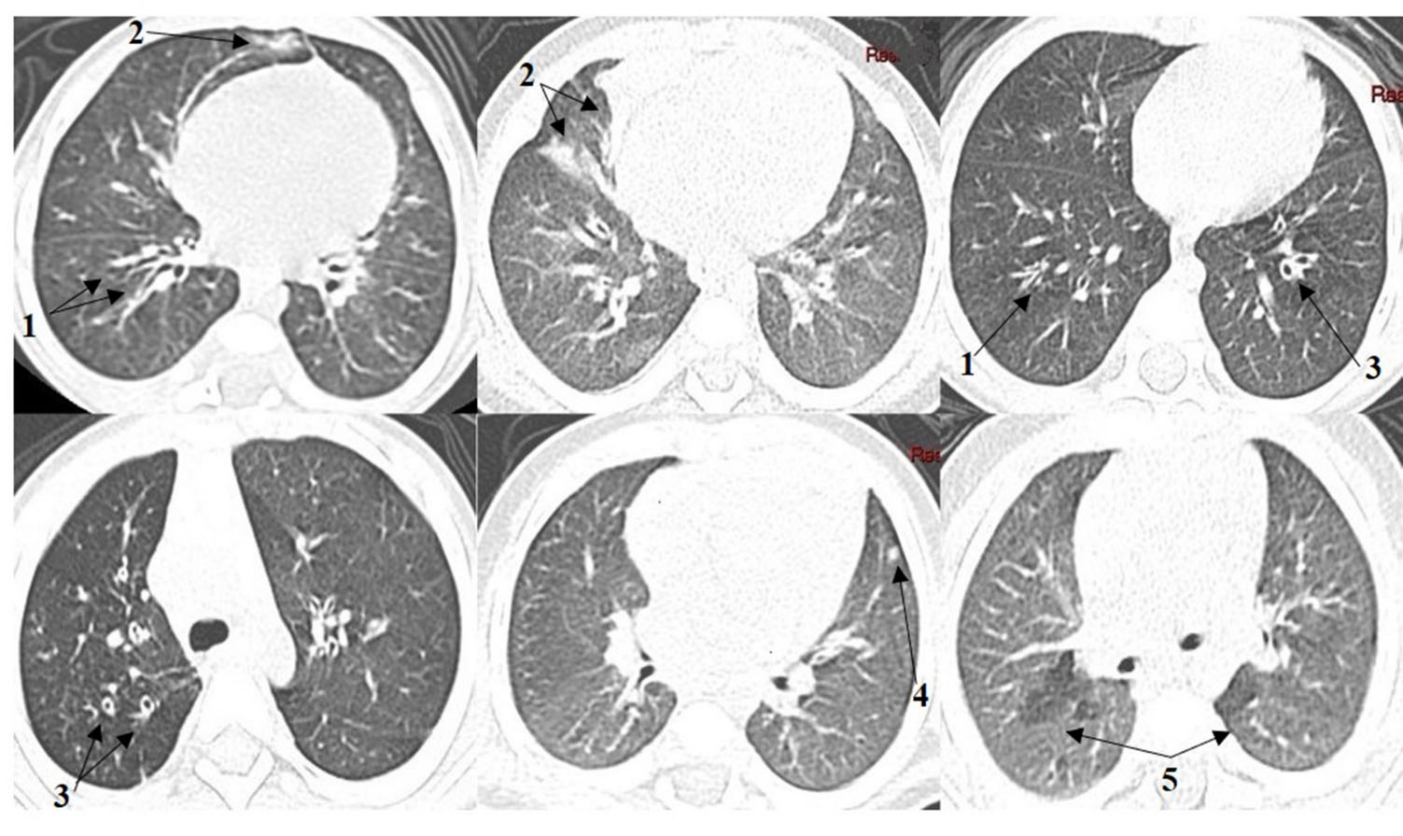
High-resolution computed tomography findings. Bronchial wall thickening (1); Collapse/consolidation (2); Bronchiectasis (3); Mucus plugging (4), Mosaic attenuation/perfusion pattern (5).

Regarding the frequencies of HRCT findings (Figure 1), bronchial wall thickening was the most common parameter (29 patients; 90.6%). Collapse/consolidation was identified in 19 of the patients (59.4%) and was subsegmental in the majority and segmental/lobar in only one patient. Mosaic attenuation/perfusion pattern was detected in 16 of the patients (50%). Bronchiectasis was identified in 12 of the patients (37.5%), and all had mild severity (score of 1) and up to five affected bronchopulmonary segments (score of 1). Other parameters such as mucus plugging (5 patients; 15.6%) and ground-glass infiltrate (3 patients; 9.4%), were less frequent. Moreover, abnormalities such as sacculations/abscesses, bullae, emphysema, acinar nodules and intralobular septal thickening were absent in these patients.

A diffuse pattern, defined as impairment in 3 or more lobes, was found for some parameters such as bronchial wall thickening in 24/29 patients; mosaic attenuation/perfusion pattern in 6/16 patients; collapse/consolidation in 4/19 and bronchiectasis in 4/12 patients. Furthermore, an assessment of the 29 patients with bronchial wall thickening revealed that 7 patients presented only bronchial wall thickening without any other abnormalities on HRCT. A total of 62% (18/29) of patients also had collapse/consolidation, 51.7% (15/29) of patients had a mosaic attenuation/perfusion pattern, and 41.4% (12/29) of patients had bronchiectasis.

## DISCUSSION

This study’s results provide outstanding insight into the theory that the early development of CF lung disease, as detected by HRCT, might begin in the first year of life. Most infants (93.8%) presented abnormal CT scans, highlighting the precocity of structural lung disease. In our study, bronchial wall thickening was present in almost all of the patients. This parameter has been frequently associated with other findings that might be related to the impairment of airway clearance (collapse/consolidation, mosaic attenuation/perfusion pattern) and inflammation, leading to permanent structural lung disease (bronchiectasis). Furthermore, a diffuse pattern of bronchial wall thickening was found in most patients. These findings at such an early age may reflect already existing airway inflammatory processes that randomly affect both lungs in stable infants with CF (28).

Moreover, this research provides clear evidence of the use of CT scans with no sedation, which prevents not only sedation-associated morbidity but also possible bias in exam findings, such as atelectasis in the posterior basal lung areas, caused by general anesthesia. Therefore, our HRCT protocol enables the use of CT as an important outcome measurement tool for young infants whose lack of cooperation may preclude other tests (12).

It is well known that CF scoring systems emphasize the importance of some abnormalities and their pathological factors; for example, mucus plugging plays a crucial role in the pathogenesis of bronchiectasis, while bronchial wall thickening reflects the presence of inflammation/infection (11,21). Long et al. (29) demonstrated that the airways in minimally symptomatic infants and young children (age 2.4±1.4 years) were thicker and more dilated than those in normal control infants, and these findings increased with age. Our study also found evidence of bronchial wall thickening in most of the patients. This corroborates the results of other studies that demonstrated that airway changes that might lead to widespread structural lung disease associated with CF begin early in life.

Our results also highlight the importance of *P. aeruginosa* in early structural lung disease, as colonization with this CF pathogen was associated with increased mean Bhalla scores compared to no bacterial colonization. In contrast, Petrocheilou et al., who evaluating 45 CF children from 6-10 years old whose diagnosis was made before 1 year of age, found that *P. aeruginosa* might not be associated with abnormal CT scans (9). These authors speculated that the absence of an association between early *P. aeruginosa* infection and abnormal subsequent chest CT findings may be attributed to aggressive treatment. Other studies including adolescent or older patients chronically colonized with methicillin-resistant *Staphylococcus aureus* or *P. aeruginosa* identified increased total modified Bhalla scores, reflecting the presence of structural lung disease (14,15). Another study on the isolation of proinflammatory pathogens (*P. aeruginosa*, *Streptococcus pneumoniae* and *Aspergillus* species) in bronchoalveolar lavage fluid showed a relationship between infection status with these pathogens and CT findings in infants (29).

Furthermore, although quantitative assessments of HRCT findings in CF patients allow accurate and reproducible analyses, they may be cumbersome and time consuming. Therefore, Oikonomou et al. evaluated five scoring systems and selected parameters that had significant correlations with pulmonary function test results. The severity of bronchiectasis and bronchial wall thickening were found to significantly worsen with time, and follow-up and might be the leading causes of clinical deterioration (30). Another study by our group that used a modified Bhalla score for HRCT findings in 41 patients found that bronchiectasis, bronchial wall thickening, and mucus plugging were present in 70% of the analyzed images (14). In our study of young children and infants, bronchiectasis, bronchial wall thickening, collapse/consolidation and mosaic attenuation/perfusion were found in more than 50% of the patients. These parameters might be considered early signs of structural lung disease and might be used as future “simplified scores” for young patients. “Simplified scores”, accounting for frequent and early abnormalities, seem faster than complete score calculations and could be easily adopted in clinical practices.

Once NBS suggests an early diagnosis, the evaluation of outcome measurements is a crucial step in maintaining high-quality CF care for this young population (31).

Despite the fact that half of our subjects presented no respiratory symptoms, more than 90% presented abnormal HRCT findings, suggesting that structural lung disease detectable on HRCT might precede the appearance of evident clinical symptoms.

In this context, the use of imaging systems, especially CT scoring systems, may help quantify structural lung abnormalities and indicate the severity of lung disease (14,16,17,20). A cross-sectional study by Mott et al. included 62 children aged one to six years who were previously diagnosed with CF by NBS and who underwent HRCT (18). Overall, 85% of the scans were considered abnormal, with bronchiectasis detected in 61% of the subjects, mucus plugging in 47% of the subjects and air trapping in 74% of the subjects. Our study showed a higher prevalence of bronchial wall thickening (more than 90%) and less frequent bronchiectasis than those in Mott et al.’s study, probably due to the difference in the subjects’ age ranges. These findings reinforce the idea that structural lung disease worsens with age; thus, its early detection might allow prompt interventions that prevent increasingly severe structural damage.

The present study on tomographic scores in patients with CF diagnosed by NBS is unprecedented in Brazil. All of the patients with CF diagnosed by NBS who were referred to this center were enrolled in this cohort and will be followed until adulthood in the same unit. The present study limitations include the cross-sectional design, as this population was part of a cohort; however, we expect to address this issue with a longitudinal prospective evaluation of disease evolution. Furthermore, as we used a single scorer to assess all the scans, there may be potential scoring bias. Nevertheless, we think this is unlikely since this scoring system has been previously validated with the same scorer in the same institution and was found to be a reproducible and reliable tool (intraobserver correlation coefficient for total Bhalla score, 0.892; interobserver correlation coefficient, 0.814) (14). We also propose a multicentric study to account for possible regional differences among a large number of patients.

In both young children and patients with mild disease, lung pathology is frequently underestimated. Thus, the early detection of structural abnormalities and their assessment with scoring systems may contribute to the identification of minimally symptomatic lung disease and allow interventions that can improve prognoses for NBS-diagnosed CF patients.

## AUTHOR CONTRIBUTIONS

Cohen RWF, Boechat MCB and Leão RS conceived, designed and coordinated the study. Folescu TW and Cohen RWF collected and analyzed the data. Marques EA and Leão RS carried out the microbiological analyses of respiratory secretions. Fonseca VM, Boechat MCB, Marques EA and Leão RS contributed to the data interpretation. Cohen RWF, Folescu TW, Boechat MCB, Marques EA, Leão RS and Fonseca VM participated in writing the draft of the manuscript. All authors read and approved the final version of the manuscript.

## Figures and Tables

**Table 1:** Anthropometric and clinical information of the subjects (Rio de Janeiro. Brazil. 2013-2016).

Features	Value
Sex (% male)	65.7% (23/35)
Birth weight (g)^a^	3056.6 (SD 452.5)
Gestational weeks at birth (weeks)^a^	38.5 (SD 1.2)
Age at CF diagnosis (months)^a^	3.8 (SD 1.9)
Weight-for-length (z-score)^a^,^b^	-0.86 (SD 1.5)
Genotype^c^	
F508del homozygous	32.2% (10/31)
F508del heterozygous	29% (9/31)
Other mutations	38.7% (12/31)
Colonization^d^	
No growth^e^	51.4% (18/35)
* Pseudomonas aeruginosa*	17.1% (6/35)
Other significant bacterial growth^f^	31.4% (11/35)
Symptoms	
No symptoms	20% (7/35)
Respiratory symptoms	45.3% (16/35)
Gastrointestinal symptoms	65.7% (23/35)
Pancreatic insufficiency^g^	77.1% (27/35)
Modified Bhalla score	3.6 (SD 2.1)

The results are expressed as the mean (SD) or % (n) unless otherwise stated.

a Mean.

b According to the World Health Organization Child Growth Standards (27).

c Complete genotype data were not available for four children.

d Based on the presence of bacteria ever isolated before HRCT.

e No bacterial growth consisted of isolation of only coliform and upper respiratory tract flora.

f Significant bacterial growth consisted of methicillin-susceptible *Staphylococcus aureus*, methicillin-resistant *Staphylococcus aureus*, *Haemophilus influenzae*, *Stenotrophomonas maltophilia*, and *Achromobacter xylosoxidans,* but never *P. aeruginosa*.

g Pancreatic insufficiency was considered according to a single assessment of low fecal pancreatic elastase levels at diagnosis if available or by the requirement for pancreatic enzyme replacement therapy at the time of examination.

**Table 2:** Correlation between subjects’ clinical/laboratory features and modified Bhalla scores (Rio de Janeiro. Brazil. 2013-2016).

	Modified Bhalla score (mean/SD or median/min-max)	*p*-value
**Age at HRCT**		
0-6 months	3.5 (1-7)	
7-12 months	3 (2-7)	0.893^b^
>12 months	3 (0-8)	
**Colonization**		
No growth	3 (0-7)^a^	
* Pseudomonas aeruginosa*	6 (2-7)^a^	0.097^b^
Other significant bacterial growth	3 (2-8)	
**Genotype**		
F508del homozygous	4.20 (SD ±1.93)	
F508del heterozygous	2.78 (SD ±1.64)	0.346^c^
Other mutations	3.58 (SD ±2.47)	
**Pancreatic insufficiency**		
Present	3.72 (SD ±2.11)	0.637^d^
Absent	3.29 (SD ±2.21)	
**Symptoms**		
Absent	3.5 (0-8)	0.837^e^
Present	3 (0-7)	
**Respiratory symptoms**		
Absent	4 (0-8)	0.782^e^
Present	3 (0-7)	
**Gastrointestinal symptoms**		
Absent	4.36 (SD ±2.62)	0.286^d^
Present	3.43 (SD ±1.80)	

a The *p*-value (Mann-Whitney test) of the comparison of no pathogens and *P. aeruginosa* was 0.048.

b Obtained by Kruskal-Wallis.

c Obtained by ANOVA.

d Obtained by Student’s t-test.

e Obtained by Mann-Whitney test.
